# Cadmium toxicity, health risk and its remediation using low-cost biochar adsorbents

**DOI:** 10.1515/biol-2025-1131

**Published:** 2025-08-11

**Authors:** Lata Rani, Jyotsna Kaushal, Arun Lal Srivastav, Shahab Abdulla, Chander Prabha, Herat Joshi

**Affiliations:** Chitkara University School of Pharmacy, Chitkara University, Himachal Pradesh, India; Centre for Water Sciences, Chitkara College of Pharmacy, Chitkara University, Punjab, India; Chitkara University Institute of Engineering & Technology, Chitkara University, Punjab, India; Chitkara University School of Engineering and Technology, Chitkara University, Himachal Pradesh, India; Analytics and Decision Support, Great River Health System, Iowa, United States of America; UniSQ College, University of Southern Queensland, QLD 4305, Australia

**Keywords:** cadmium, adsorption, biochar, mechanism, water treatment

## Abstract

Cadmium induces toxicity to both flora and fauna, even when it is present in trace amounts. Electroplating, pigments, smelting, mining, alloy production, plastic, cadmium–nickel batteries, fertilizers, pesticides, paint, synthesis of dye, textile operations, and refining sectors all release cadmium into the aquatic environment. “Solvent extraction, adsorption, ion exchange, and precipitation” are a few strategies for removing cadmium. Biochar is an inexpensive and sustainable adsorbent that has proven to be an efficacious adsorbent for the recovery of Cd(ii) from water. This study discusses the toxicity of cadmium as well as some recent developments of pristine biochar and modified biochar for the elimination of cadmium (Cd) from aqueous solution.

## Graphical abstract

**Figure:**
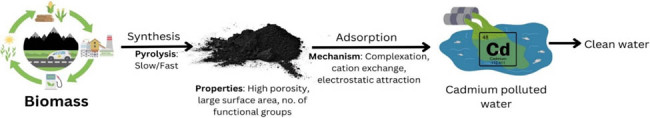

## 1 Introduction

Heavy metal contamination in water has become a major concern for the community, industries, and government agencies [1]. Cadmium (Cd) is released into the environment by a variety of anthropogenic and geological processes, including the burning of fossil fuels, the production of batteries, pigments, and fertilisers, the making of iron, steel, and cement, mining, and the stabilisation of PVC products. Geogenic processes include rock weathering, soil particles, volcanic eruptions, forest fires, and sea salt [2]. Because exposure to it through contaminated water can result in kidney and liver dysfunction, damage to the haematopoietic system, bone, testicles, and adrenal glands, neurological disorders, pulmonary oedema, and chronic itai-itai disease, it is categorised as a hazardous and priority pollutant. Additionally, the International Agency for Research on Cancer has classified it as a human carcinogen (group I) [3]. Consequently, a cadmium level limit of 0.005 mg/L has been set for drinking water by the Argentine Food Code and the US Environmental Protection Agency [4]. One of the difficulties humanity faces in ensuring sustainable growth and environmental safety is protecting the environment and human health. Membrane separation, ion exchange, and chemical precipitation are the top three standard methods for extracting or recovering cadmium from wastewater. Regretfully, these procedures can be costly to run and materially expensive, and they can generate a large amount of sludge [3]. Adsorption is a surface phenomenon in which mass is transferred by weak physical forces, such as van der Waals forces, or strong chemical interactions, such as covalent bonds, between a liquid (or gas) phase containing the adsorbate and a solid phase containing active adsorption sites [5]. The best adsorbent one that is inexpensive, has a large surface area, a high adsorption capacity, mechanical stability, and is easily regenerable has not yet been discovered, despite adsorption’s recent promotion as an economical, effective, and user-friendly technology. The interaction between the adsorbent and the contaminant, and consequently, the efficacy of the adsorption process, is influenced by various factors, including pH, temperature, ionic strength, contact time, initial adsorbate concentration, and adsorbent dose [6].

Activated carbon is used as an adsorbent in many industries, but its primary drawback is the high cost of manufacture and regeneration [7]. A large amount of solid waste is composed of agro-industrial waste, and because effective treatment is expensive, managing this garbage can be difficult in developing nations. These wastes gain economic value from their reuse, which thus acts as a motivator for the agro-industrial sector [8]. These wastes, which include banana peels [9], lentil husks [10], eggshell powder [11], and peanut shells [12], have been explored as Cd(ii) adsorbents since they are abundant, affordable, and readily available in the area. The affinity and selectivity of these materials for cadmium and other heavy metals are facilitated by the presence of binding groups, such as ether, carbonyl, and hydroxyl, on their surfaces.

The production of biochar, a carbonised form of biomass generated under oxygen-limited conditions and moderate temperatures (700°C), is another option for the reuse or management of agro-industrial waste [13]. Because of its high efficacy and lack of need for sophisticated activation techniques, this material has recently been suggested as an adsorbent for the removal of heavy metals [14]. Biochar’s structure differs from activated carbon in that it lacks activation and contains both carbonised and noncarbonised fractions with micro-, meso-, and macropores, a large surface area, and oxygen functional groups and minerals. These characteristics improve biochar’s adsorbent properties compared to its source biomass.

On the basis of a literature survey, it was found that only a few review articles have been published previously on the application of biochar for the removal of cadmium from water [15,16]. However, there are several reviews on the role of biochar in the removal of heavy metals from water [17,18,19,20,21,22,23,24]. The recently published review focused on the application of the modified biochar for the remediation of cadmium from the water. To the best of our knowledge, limited information is available on the critical evaluation of biochar for the adsorptive removal of Cd(ii) from water and wastewater. Additionally, the relative performance of pristine biochar versus modified/composite biochar for heavy metals (or cadmium) removal from aquatic systems was reported with limited information. Critical discussion on the sustainability of the adsorptive removal of heavy metals (or cadmium) from water and wastewater, potential regeneration methods to enhance biochar lifecycle, and management of spent biochar was not considered in earlier reviews. Further, the previous reviews did not critically discuss the toxicity caused due to the exposure of Cd(ii).

Therefore, the main objective of this review paper is to critically analyse the recent developments in the application of pristine and engineered biochar for the removal of Cd(ii) from water. The technologies used to produce biochar and the characterisation of biochar using both conventional and cutting-edge technologies for desired environmental remediation applications are presented. The influence of various factors such as water chemistry, adsorbate concentrations, the presence of competing ions, adsorbent (biochar) characteristics, and environmental parameters (e.g. temperature) on the Cd(ii) adsorption performance is discussed. Insights into potential adsorption mechanisms, sorption isotherms, kinetic behaviour, and thermodynamics of the adsorption phenomenon are provided. This review identifies key knowledge gaps and points out research directions that should be considered in the future since these recommendations would be helpful for the scale-up of the biochar-based adsorption technology for optimum decontamination of Cd(ii) contaminated water and wastewater systems.

## 2 Toxicity of cadmium on human health

**Figure 1:**
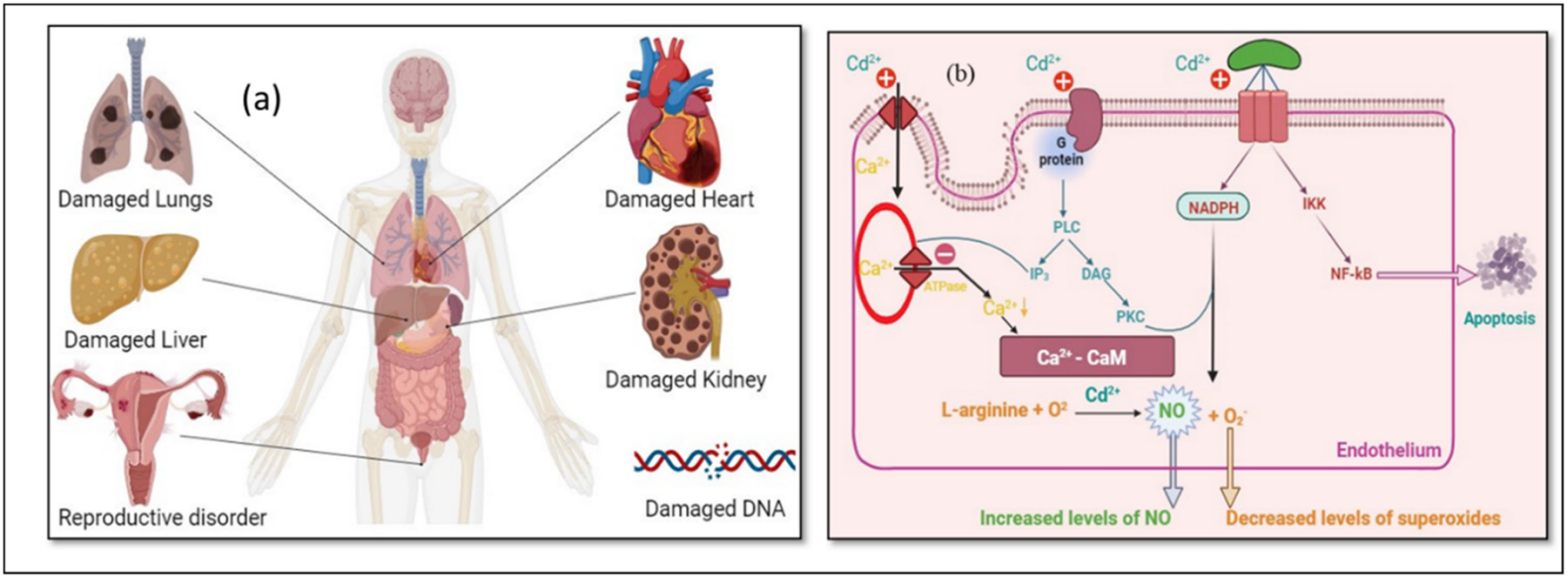
(a) and (b) Toxic effects of cadmium on human health and mechanism.

**Figure 2:**
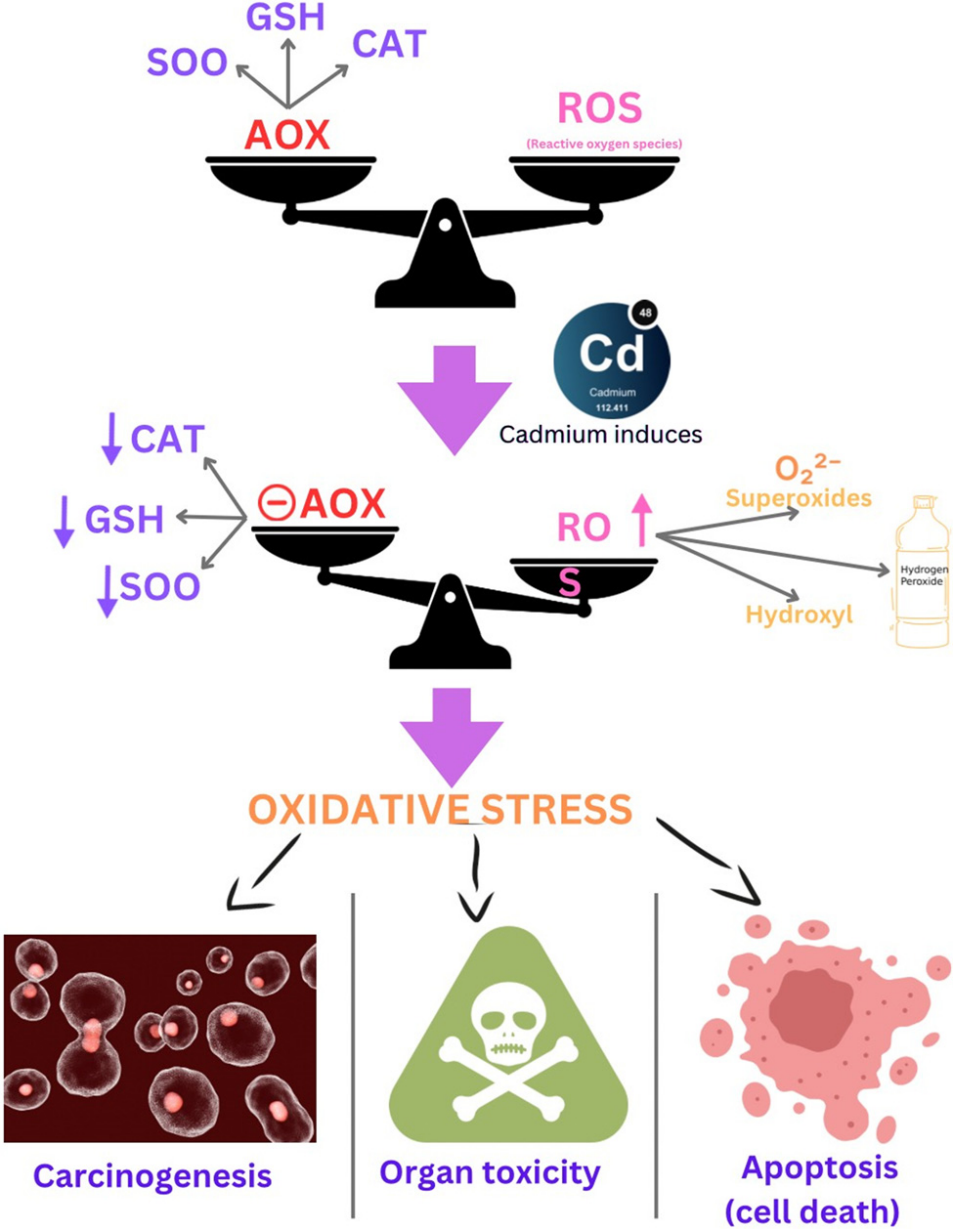
Mechanism of toxicity of cadmium in the human body.

Chronic accumulation of cadmium results in multiorgan toxicity, primarily targeting the kidney, skeleton, liver, and nervous system [25]. Among these, cadmium poisoning is especially dangerous for the neurological system. According to Viaene et al. [26], cadmium can raise the risk of peripheral neuropathy, disturbed balance, and subpar visuomotor ability. According to Li et al. [27], exposure to cadmium is linked to decreased concentration, worse cognitive function in the elderly, and worse learning outcomes in children. Additionally, neurodegenerative disease abnormalities seen in amyotrophic lateral sclerosis, Parkinson’s disease, and Alzheimer’s disease have been linked to cadmium exposure [28]. There are several ways that cadmium causes neurotoxicity [29]. The toxic influence of cadmium on the health of humans and its mechanism are shown in Figures 1(a), (b), and 2.

### 2.1 Kidney damage

The primarily targeted organ for cadmium toxicity is the kidney. Cadmium accumulates in the proximal tubule’s SI section, where it causes oxidative injuries in the mitochondria and transports proteins that cause tubular cell death [30]. The cadmium accumulation in the tubule area of the kidney is almost 30% of the body, and the injuries in the tubule are linked to the amount of cadmium that does not bind to metallothionein (MT) [31].

The toxicity of cadmium-engendered defects in the metabolism of vitamin D in the kidney leads to venomous effects on the bones. This effect is directly related to cadmium mutilation of calcium absorption in the gut as well as disruption of collagen metabolism, both of which lead to osteoporosis and osteomalacia [32].

The degree of kidney damage caused by cadmium is contingent upon the accumulation level of Cd–MT in organs, primarily affecting the primary tubules and showing proteinuria symptoms. Long-term exposure to cadmium is indicated by detectable concentrations of cadmium in urine (Cd-U), generally estimated in accordance with creatinine, signifying kidney burden. Symptoms may affect multiple organs, with kidneys being the most impacted. In the renal cortex, cadmium critical concentration can reach up to 200 mg/kg in exposed individuals, while urinary levels range from 0.889 mol/L (10 g/g creatinine) to 1.333 mol/L (15 g/g creatinine). The biologically permissible level of Cd in urine is 0.0445 mol/L (5 g/g creatinine). The estimated prevalence of elevated urine cadmium range (>2 g/g creatinine) is 2.3% of the US population. Proteinuria may persist as a prominent symptom, followed by glycosuria, aminoaciduria, elevated urinary excretion of calcium and phosphorus as well as elevated creatinine level. Often, exposure only results in Cd-induced toxic nephropathy [33].

Significant threats are also associated with the slow excretion of cadmium from the body, which mostly occurs through sweat, saliva, faeces, and urine. Urinary cadmium excretion occurs in three stages: (1) involves Cd accumulation at the renal cortex, with urinary excretion proportional to kidney content, reflecting past exposure; (2) marked by large exposure and MT depletion, incorporates exposure from the past and the present; and (3) characterised by renal tubular damage, results in significant urinary cadmium excretion, reflecting ongoing exposure and kidney elimination [34,35,36].

### 2.2 Cadmium nephrotoxicity

Once Cd enters the bloodstream through absorption from the lungs and/or intestine and conjugates with the blood protein albumin, it is carried to the liver, where it has a detrimental effect on the heart, kidneys, and liver, among other prominent organs. Cd and MT mix in hepatic cells to create Cd–MT complexes, which are filtered by the kidneys after traveling there via the bloodstream [37]. The primary organs in which chromium build-up damages glomeruli and proximal tubules are the kidneys. Renal Cd is indicated by the presence of Cd in urine, which is a biomarker for renal Cd accumulation and chronic exposure [38]. Urinary creatinine of 0.67 µg/g signifies injury to the tubules, while 0.8 µg/g implies damage to the glomerulus [39].

There are numerous main pathways through which cadmium may damage the neurological system. First, entering the body by eating, cadmium eventually finds its way into the bloodstream and the central nervous system through the blood–brain barrier (BBB). Alternatively, cadmium can be inhaled and build up in the olfactory bulb. Because cadmium weakens the BBB, there is a greater chance of more cadmium entry. The transporters for calcium and zinc can then allow cadmium to enter cells and cellular compartments. Intracellular cadmium has the potential to significantly impair mitochondrial function, change neurotransmitter signalling, and seriously impair glycogen metabolism, all of which raise the risk of neurodegenerative consequences. Cadmium causes neurotoxicity in several ways [40,41].

### 2.3 Cancer

According to Chen et al. [42], mercury is recognised as a human carcinogen that can lead to cancer in a number of organs, such as the kidneys, breast, prostate, and lungs. In addition, it produces reactive oxygen species (ROS) like superoxide, nascent oxygen, hydrogen peroxide, and hydroxyl free radicals, which can be genotoxic and cytotoxic [23]. ROS causes severe damage to DNA, which leads to mutations if repairs are not made. Replication-born errors are caused by the incorrect base being incorporated and the proofreading system malfunctioning. ROS have the ability to damage proteins, and if these proteins are involved in DNA replication, replication-born mistakes may ensue. This oxidative stress caused by metals is also harmful to various human organs and tissues [43].

Several studies have determined that cadmium plays a major role in the development of a variety of cancers. Evidence connecting occupational Cd exposure to lung cancer provides significant support for this view. The lungs are one of the most vulnerable organs to cadmium exposure in humans. Furthermore, a number of studies have found a link between exposure to cadmium and the emergence of kidney and prostate cancers. There is compelling evidence of cadmium’s direct effect on the human prostatic epithelium since recent research indicates that exposure to cadmium *in vitro* can cause cancer transformation of prostate epithelial cells. Furthermore, two recent investigations have brought attention to the connection between occupational Cd exposure and renal cell carcinoma. A more conclusive link between cadmium exposure and the emergence of prostatic or renal cancer may be possible with the use of genomic fingerprinting, which has been used to find signatures for a variety of hepatotoxic substances. A small number of studies have linked occupational cadmium exposure to cancers of different organs, such as the stomach, liver, haematopoietic system, and bladder. Given the extremely deadly nature of pancreatic cancer, the latest studies claimed that cadmium exposure might be connected to the development of pancreatic tumours in humans is both persuasive and significant [44,45,46].

### 2.4 Cardiovascular disorder

It was detected from outcomes of the *in vitro* investigations that cadmium toxicity was involved in the disturbance in the function of endothelial and carotid intima-media thickness. Although *in vivo* atherosclerotic plaque formation has been supported [47], subsequent cadmium “intoxication,” endothelial ailment at the start of “cardiovascular ailment,” loss of endothelial cell structure that led to the death of cells, and some minor thrombogenicity were observed. These outcomes supported the assumption that cadmium toxicity was associated with cardiovascular disorders as well as heart attack [48]. Furthermore, different epidemiologic studies support the risk of hypertension associated with cadmium toxicity. The endothelial cell injury caused by this promotes the production of cytokines. It could enhance the rate of cardiovascular mortality.

Cardiovascular muscles get injured by cadmium before the liver and kidneys. Heart muscles need a constant supply of ATP, which is produced by the oxidative degradation of glucose in mitochondria, in order to operate properly. According to Azevedo et al. [49], cadmium stress has been shown in rats to obstruct the pyruvate-malate route of mitochondrial respiration, which results in the death of heart cells or a variety of cardiovascular diseases. As of now, no particular treatment has been suggested for Cd toxicity [50]. As a result, we must act quickly to protect and preserve our environment by reducing the levels of heavy metal contamination, or else the situation will quickly be out of control. Cadmium inhibits the formation of “endothelial nitric oxide synthase” as well as acetylcholine-induced vascular relaxation induced in hypertension [51]. It would stimulate the generation of cytokines and cause damage to endothelial. These mechanisms could induce atherogenesis, and longer-term exposure would elevate the occurrence of peripheral arterial syndrome [52]. The rate of death due to cardiovascular disorders induced by exposure to cadmium is very high.

### 2.5 Reproductive disorder

In rats, cadmium seems to obstruct the ovarian steroidogenic pathway. Piasek and Laskey [53] assessed the direct effects of cadmium exposure *in vitro* on rat ovarian steroidogenesis. Progesterone and testosterone production was the most impacted. It has been observed that ovarian progesterone production is stimulated by low dosages of cadmium and inhibited by high dosages. Low birth weight and an increase in spontaneous abortions are linked to maternal exposure to cadmium [54]. There is also some evidence that, both *in vivo* and *in vitro*, cadmium is a strong nonsteroidal oestrogen. Research conducted on rats revealed that cadmium causes increased uterine weight and accelerated mammary development [55].

### 2.6 Itai-Itai disorder

A number of investigations conducted in the twentieth century, such as those involving workers exposed to dust and fumes contaminated with cadmium, demonstrated a link between cadmium poisoning and bone injury [56]. Additionally, it may be demonstrated that cadmium is linked to Itai-Itai cases. Patients with this condition exhibit a variety of symptoms, including low bone mineralisation, a high fracture rate, an increased risk of osteoporosis, and excruciating pain related to the bones. In the 1940s, an epidemic of the Itai-Itai sickness was noted in the Jinzu River basin in Japan. In this instance, a study revealed that patients exhibited the typical symptoms following consumption of rice cultivated on heavily cadmium-polluted irrigation fields. Severe bone decalcification and pseudofractures indicative of osteomalacia were also detected. The bulk of the patient collection consisted of postmenopausal women, which raised criticisms for this study [57].

Honda et al. [58] established additional evidence that cadmium poisoning is causally related to bone ailments. They might explain a negative relationship between urine cadmium levels and the STIFF index, an ultrasonography technique for determining bone density [59]. Similar results were obtained in the OSCAR Study, which involved 1,021 participants from southern Sweden. Here, it was found that there was a strong negative link between poor bone mineral density and urine cadmium content, particularly in people 60 years of age and older. Furthermore, it was discovered that people exposed to cadmium had a higher incidence of forearm fractures. Participants in this research were either employees of the battery company or locals living in the town near the plant. A group of individuals who had not been exposed is included as a reference group.

## 3 Remediation techniques

**Figure 3:**
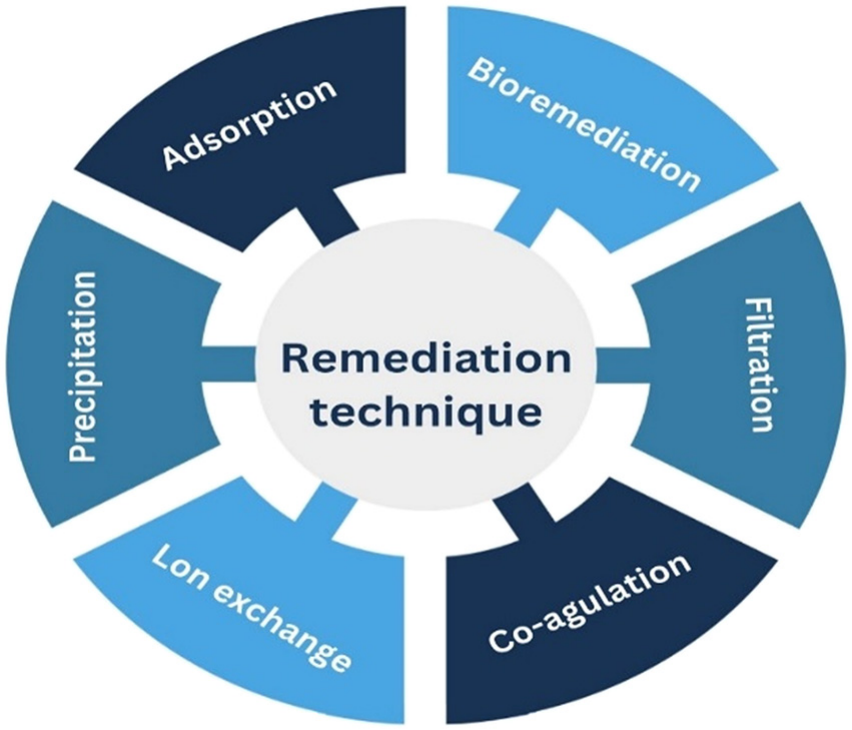
Methods utilised for the recovery of cadmium from waste water.

As demonstrated in Figure 3, numerous procedures were applied to efficiently remove Cd(ii) from aquatic environments; for instance, “ion exchange, flotation, electrodialysis, co-precipitation, membrane filtration, coagulation, and adsorptions” [60].

Heavy metal removal from wastewater has been accomplished by the widespread use of flotation. The primary flotation methods for removing metal ions from solution include foam flotation, dissolved air flotation, ion flotation, precipitation flotation, and adsorbing colloid flotation [60]. The process of creating foam in foam flotation involves using a surfactant to make a non-surface-active substance surface-active. This produces a product, which is then removed by bubbling a gas through the bulk solution. Foam flotation has various unique benefits when dealing with diluted wastewaters that contain heavy metals in parts per billion or parts per million ranges.

Ion exchange methods are widely used in heavy metal removal due to their major advantages, which include high treatment capacity, high removal efficiency, and quick kinetics, as reported by Kang et al. [61]. We can selectively remove a particular metal ion from a solution or remove all metal ions from it using ion-exchange [62]. Synthetic resins are the most favoured material among those employed in ion-exchange procedures because they are very good at eliminating heavy metal ions from solution. In the ion exchange technique, the volume of sludge produced is low due to the selective recovery of metal ions [63]. However, it has many disadvantages, like its high cost and time consumption.

Heavy metals are also removed from wastewater by coagulation and flocculation, which are followed by sedimentation and filtration. Destabilising colloids by eliminating the forces holding them apart is known as coagulation. In conventional wastewater treatment processes, a variety of coagulants are commonly employed, including aluminium, ferrous sulphate, and ferric chloride. These coagulants effectively remove wastewater particulates and impurities by neutralising particle charge and entangling impurities in the precipitates that form, which are amorphous metal hydroxides [60].

There are many advantages of “membrane filtration” compared to other methods, like high removal efficiency, being environment friendly, and saving energy, but the cost of this method is very high [64]. The precipitation technique is widely used for the recovery of the cadmium ion from the “water,” and it is carried out in a column for pilot-scale experiments. This technique is simple to use and less expensive.

Chemical precipitation is by far the most popular method for removing heavy metals from industrial wastewater; roughly 75% of electroplating facilities use precipitation treatment treating their wastewater with hydroxide, carbonate, or sulphide precipitation treatment, or various combinations of these treatments [65]. Chemicals and heavy metal ions combine during precipitation processes to create insoluble precipitates. Sedimentation or filtration can be used to remove the precipitates from the water. After treatment, the water is appropriately released or recycled [60].

Electrodialysis is a commonly used technique to remediate cadmium ions from water. This technique has several merits, including elevated efficiency, simple operation, along selective removal of heavy metals, although it also suffers from many shortcomings, like being expensive and producing highly hazardous waste. Further, bioremediation is another effective approach to recovering heavy metals from wastewater, and Cd(ii) “remediation” has been accomplished via the interaction of microorganisms with heavy metal ions. Although it is lower expenditure, environmentally friendly, requires less technology, and produces no waste, it has some limitations, such as complex operation and high specificity, which necessitates suitable environmental conditions and proper contaminated nutrition [66].

Adsorption is the most favourable of these approaches owing to its easy operation, lower cost, and ability to produce fewer toxic wastes. As the nature of most of the adsorption process is reversible, the adsorbent could be recovered only via an appropriate process of desorption, which could be utilised several times [67]. Furthermore, the desorption methods are more efficient, have a simpler operation, and are less expensive. As a result, adsorption has emerged as the superior method for extracting Cd(ii) from aquatic systems.

Adsorption is the most effective way for recovering Cd(ii)-contaminated water. Adsorption is the way of transferring a “substance from the liquid phase to the solid surface and substance linked by physical or chemical bonding” [68]. For the recovery of Cd(ii) from the aqueous phase, a variety of adsorbents are used, including activated carbon, nanoparticles, macromolecules, and biomass. The primary drawbacks of these adsorbents include their high cost, poor selectivity, and limited efficiency. Researchers are currently searching for adsorbents that are inexpensive, highly effective, and environmentally favourable. The researchers were drawn to biochar because of its strong Cd(ii) adsorption capacity. Agro-waste and industrial wastes would be used for making biochar [69].

## 4 Biochar

By heating biomass, “particularly waste from agricultural and industrial” in an air-restricted environment (pyrolysis) generates a solid and carbon-rich by-product, which is known as biochar. First, it was thought to be a substance that could store carbon and so help in reducing global warming [70]. Owing to its distinct surface chemistry, which includes higher aromaticity, multiple functional groups, higher surface area, and high alkalinity [71], biochar was subsequently known to have higher immobilisation abilities for heavy metals in water. The availability of biochar in water treatment has enormous potential due to its many additional advantages, such as waste reuse, low carbon footprint, and affordability [72]. According to Lehmann and Joseph, “a high carbon-content product was generated when biomass, for example, leaves, manure, and wood, had been heated in the presence of an inadequate supply of oxygen.” Biochar is defined as “a porous carbonaceous solid substance with physical and chemical features that make it an acceptable for sustained safe supply of carbon via the thermochemical operation in the exhausting environment” [73].

According to Lehmann and Joseph [70], biochar differs from charcoal in the way it is utilised. Biochar may be used for controlling environmental pollution, and charcoal is used as a source of energy and fuel. Biochar and hydrochar are strongly associated with each other, but hydrochar differs in the way of production by the hydrothermal process [74].

### 4.1 Derivation of biochar

**Figure 4:**
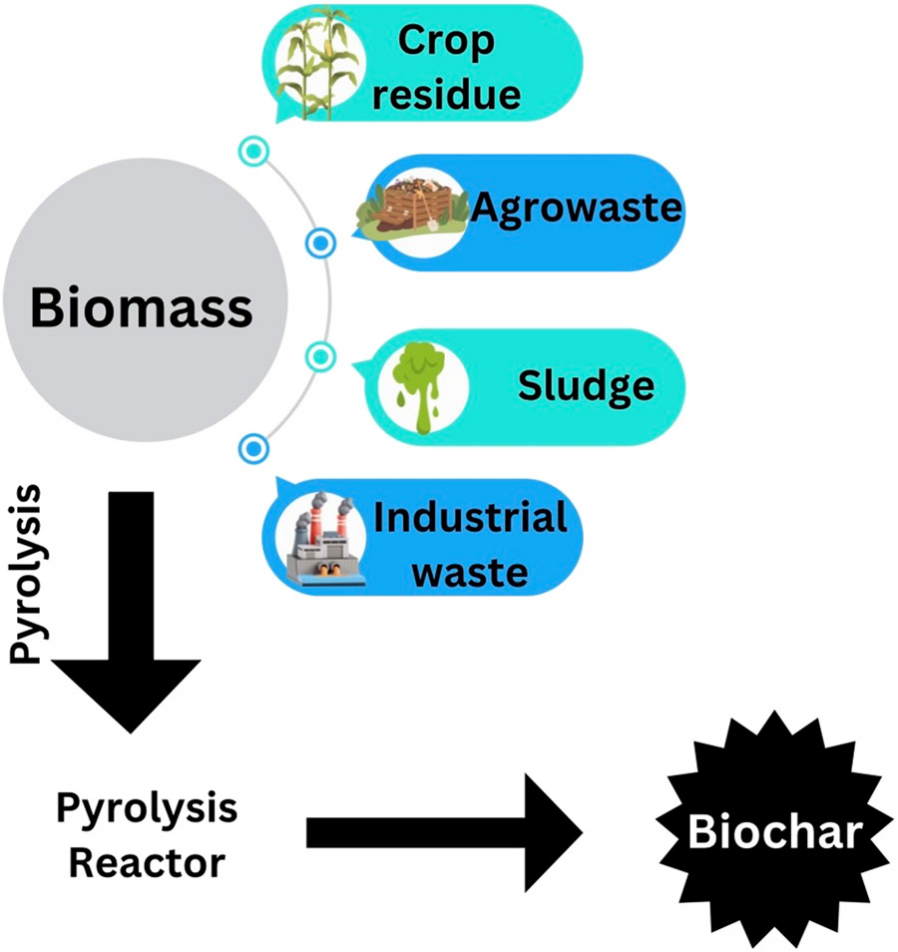
Synthesis of biochar by the pyrolysis method.

Biochar is characterised by physical and chemical parameters, as shown in Figure 4. It is observed that the alteration in the physical characteristics of the biochar is clearly seen when biochar is in a pelleted or granular arrangement. The melting rate of fine biochar is greater than that of pellets. The uniform addition of pellet form of biochar is quite difficult compared to amending soil as well as biochar of improved quality. The life span of biochar having greater amounts of ash is lesser in the natural system of soil due to a greater rate of deterioration [75].

### 4.2 Pyrolysis

As shown in Figure 4, the most familiar procedure for producing biochar was pyrolysis. Pyrolysis is divided into two classes, slow and fast, according to the residence time and heating rate. By heating the biomass at a lower rate for a longer residual period, biochar is produced during slow pyrolysis (conventional carbonisation). Since ancient times, this process has been employed to make charcoal. Fast pyrolysis generated charcoal at a faster rate and for a minuscule period of time. The yields of biochar and bio-oil produced by these two processes are different; slower pyrolysis produces greater yields of biochar [76]. Based on process criteria like residence time, type of feedstock, rate of heating, and temperature, biochar produced by slow pyrolysis has certain physicochemical characteristics and yields [77].

### 4.3 Characteristics of biochar

**Figure 5:**
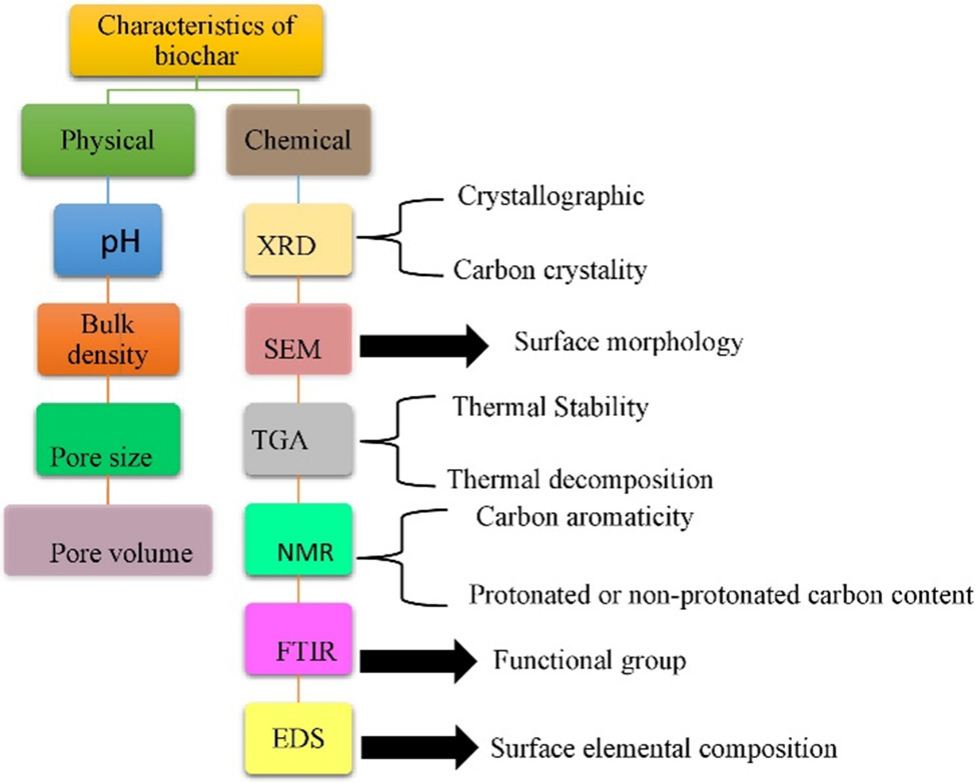
Characterisation techniques for biochar.

Biochar is characterised by physical and chemical parameters. The characterisation of techniques for biochar is shown in Figure 5. It is observed that the alteration in the physical characteristics of the biochar is clearly seen when the biochar is in a pelleted or granular arrangement. The melting rate of fine biochar is higher than that of pellets. The uniform addition of the pellet form biochar is quite difficult as compared to amending soil, as well as biochar of improved quality. The life span of biochar with greater amounts of ash is shorter in the natural system of soil due to a greater rate of deterioration [75].

The extraction of biochar at a lower pyrolysis temperature gives a higher yield and has a greater amount of volatile matter compared to biochar generated at elevated temperatures. With the increase in temperature, the yield of biochar, as well as the amount of volatile matter, decreased. Additionally, the yields of the biochar and the value of volatile matter are changed with the “nature” of the feedstock. For example, non-woody biochar shows lesser alteration in the amount of volatile value than woody biochar when pyrolysis temperature increases from 400 to 800°C [78]. Woody biochar consists of a higher amount of volatile matter at lower temperatures because woody feedstock contains lignin that would hinder the pyrolytic breakdown at a pyrolysis temperature of 400°C, although it cannot hinder the process when the temperature is higher than 950°C for the assessment of the amount of ash. However, biochar obtained from the rice material has a higher amount of ash matter at almost all temperature ranges, and it might result in partial alteration in the constitution enhanced by the interaction between inorganic and organic components by the process of feedstock to synthesise biochar, which has an ash content greater than 20% [78].

Chemical characterisation of individualistic feedstock variety and, consequently, biochar obtained from the feedstock have played a vital role spatially as well as temporarily [79]. The manufacturing of biochar is frequently evaluated by means of alteration that would take place in the elemental concentration of carbon, hydrogen, nitrogen, oxygen, and sulphur and related ratios [80]. When a particle sample is carbonised, fixed carbon remains as a solid-combustible residue, and volatile components are removed. Therefore, it is utilised for the estimation of carbonaceous substances, which are produced from the solid sample. To determine the maturation and the degree of aromaticity, generally, O/C and H/C (hydrogen/carbon ratio) ratios are utilised. Yu et al. [81] employed biochar as a catalyst in the transesterification of “canola oil.” They observed that during the elemental analysis of biochar with elevation in the pyrolysis temperature, the “ratios” of H/C and O/C would decline. It was believed that the amount of pyrolysis and the degree of oxidative modification in water and soil are assessed using the elemental ratios of “O/C, O/H, and C/H” [81]. Biochar synthesised by the “pyrolysis” method is characterised by elemental analysis, zeta potential, CEC analysis, and pH measurement of colloids of biochar by various processes [69]. As apparent in earlier reports, the biochar prepared by fast pyrolysis has a fine structure because of quick modification in a fast pyrolysis reactor as well as holding a high grade of thermal mutation with lower carbon and hydrogen ratio compared to gasification or slow biochar [82]. Other characteristics are surface area [70] and pH.

Biochar with a high porosity and larger surface area would often have better sorption properties. When there is an increase in water loss during the dehydration process, the pyrolysis process forms the porous surface in biochar. The International Union of Pure and Applied Chemistry states that there are three possible types of pores in biochar: micro (<2 nm), meso (2−50 nm), and macro (>50 nm). Regardless of the pesticide molecules’ charges or polarity, the biochar with smaller pores is unable to adsorb them. Scanning electron microscopy (SEM) was used to characterise the size of the pores in biochar. Temperature has a significant impact on the creation of biochar, although surface area is the primary factor in determining biochar sorption capacity. Raw materials that have been treated or not may have different surface areas. Activated carbon has a larger surface area in the market. Lacking an activating mechanism, the biochar generated has a lower surface area and is less porous [83]. Therefore, the activation procedure is used during the manufacturing of biochar to improve the surface area and porosity of the material. The process of activation may entail both chemical and physical activation.

The economics of biochar systems and their effects on energy and climate change were estimated using life cycle assessment (LCA). In contrast, gasification technology has superior economic qualities compared to the other two technologies (pyrolysis and torrefaction), LCA. The study revealed that process and feedstock have a major impact on the characteristics and pace of production of biochar. Applications of biochar in agriculture have the advantageous effect of lowering emissions and sequestering carbon, particularly when it comes to minimising the carbon footprint of farms. Nonetheless, the intricacy of the manufactured feedstock’s composition and the discrepancy between the characteristics of biochar and the intended use are seen as possible dangers.

### 4.4 Environmental impacts

LCA has been widely used to evaluate these effects, with the majority of research concentrating on the possible decrease in greenhouse gas emissions, which is one of the biochar’s main benefits. Furthermore, recent studies have conducted comparisons among metal-based catalysts, activated carbon, and biochar [84,85]. Gallego-Ramírez et al. [86] discovered that the environmental effects of gasification-based production of raw and Fe-modified biochars are mostly influenced by the energy source and the gasification process.

LCA is an essential instrument for examining the possible environmental effects of goods, procedures, or activities. LCA offers thorough insights into the ecological effects of a system or product by assessing the full life cycle, from the procurement, production, and use of raw materials to disposal or recycling [87,88]. To properly conduct an LCA, comprehensive information on energy, materials, and environmental outputs, including the effects on soil, water, air, and waste, must be gathered. From the procurement of raw materials to the end of life, these data are examined using certain approaches to evaluate environmental consequences and pinpoint areas in need of improvement.

### 4.5 Sustainability

**Figure 6:**
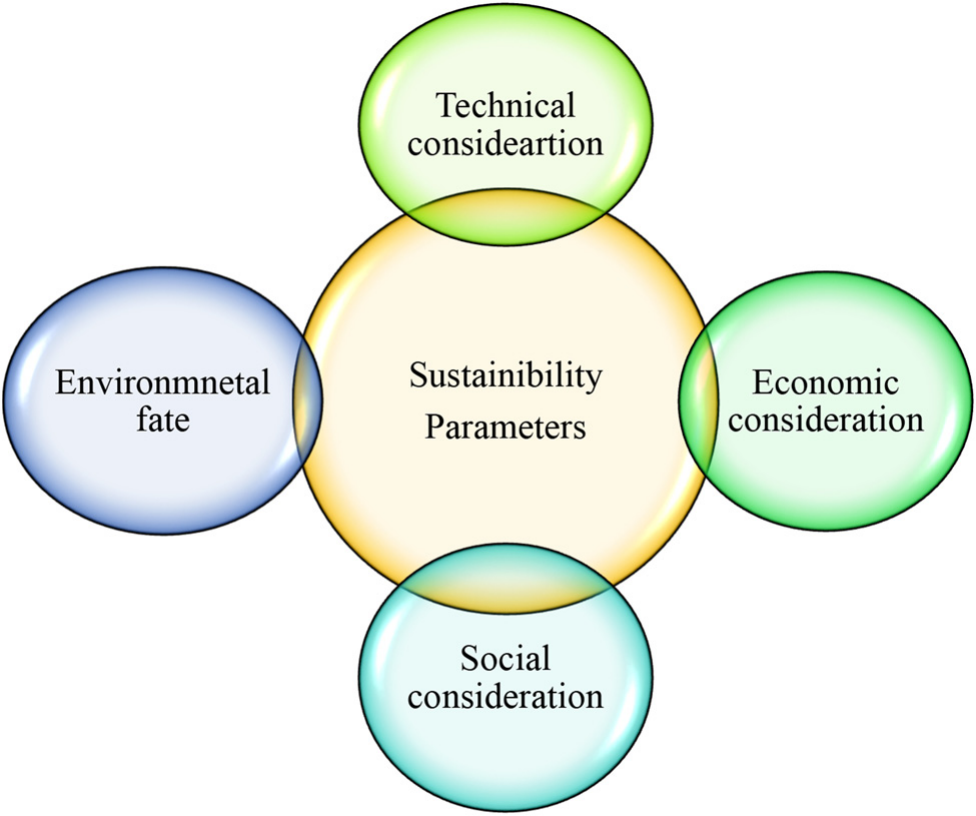
Parameters determining the sustainability of the biochar.

The parameters determining the sustainability of the biochar are technical considerations, environmental fate, economic considerations, and social considerations, as shown in Figure 6.

## 5 Application of different types of biochar to remove cadmium

### 5.1 Pristine biochar

Xu et al. [89] derived a biochar from dairy manure and rice husk to extract “Cu, Zn, Cd, and Pb” from water. All four metals are removed more efficiently by dairy manure biochar in comparison to “rice husk biochar.” In dairy manure biochar, heavy metals are adsorbed on the ionised hydroxyl-*O*- groups and also formed precipitate by reacting with PO_4_^3−^ or CO_3_^2−^; on the other hand, in the case of rice husk biochar, only adsorption occurred on ionised phenolic O^–^ groups. Therefore, this study indicated that minerals, for example, PO_4_^3−^ and CO_3_^2−^ and “functional groups,” play an amazing role in the adsorptive elimination of these “heavy metals” (Cha et al., 2016). Kim et al. (2015) have prepared a biochar from giant Miscanthus by slow pyrolysis in the temperature range of 300–600°C within a 100°C interval. At temperature >500°, the surface area and pH of the biochar increased, which led to an increase in the adsorptive potential of Cd(ii). The detailed summary of pristine biochar applied for the remediation of cadmium and the aqueous phase is presented in Table 1.

**Table 1:** Adsorption capacities of pristine biochar for the adsorption of Cd(ii)

Biochar as adsorbent	Pyrolysis temperature (K)	Initial concentration (mg/L)	Adsorbent dose (g/L)	pH	Adsorption capacity (mg/g)	Isotherm model	Kinetic models	Contact time (min)	Mechanism	References
Pine bark	673	60	10	4–5	0.34	Freundlich	First	1,440	Ion exchange	[96]
Oak wood					0.37					
Oak bark					5.4	Langmuir	First			
Carbon F-400					8					
MBCG	773	0.3	20	5	84.76	Langmuir	Pseudo- second	4,320	Electrostatic	[97]
Rape straw	873	20	1.25	5.5	81.10	Langmuir	Pseudo-second	720	Chemosorption	[98]
Banana peel	873	200	0.5		103.22	Freundlich	Pseudo-second	120	Electrostatic	[99]
						Langmuir				
Cauliflower	873	200		3–8	70.83	Freundlich		120		
						Langmuir				
W-BC	873	5	2	5	18.06	Langmuir	Pseudo-second	40	Complexation	[100]
C- BC					18.8					
B- BC					19.12					
APBC	<473	—	0.02	—	23.54	Langmuir	Pseudo-second	—	Complexation	[101]
OPBC		—		—	19.04			—		
HMO-BC	<973		3	3–7	22.3	Freundlich	Pseudo-second	120	Complexation	[102]
BWBC	973	50	1	5	11.63	Langmuir	Pseudo-second	240	Complexation	[91]
LDH-Kiwi biochar	773	50	0.02	6.5	30.96	Langmuir	Pseudo-second	1,440	Complexation, isomorphic substitution, and chelation	[92]
CSB	773	50	1	7	52.1	Langmuir	Pseudo-second		The formation of chemical bonds between Cd ion and the adsorbent functional groups)	[103]
PSB					44.44					
PWB					8.07					
Mg-BC	723	50	0.01	5	178.97	Freundlich	Intraparticle diffusion	1,440	Precipitation, cation π-binding, complexation, and ion exchange	[104]

Regmi et al. [90] synthesised biochar from switchgrass by the hydrothermal carbonisation method. The authors analysed the influence of adsorbate dose, pH, and equilibrium time. The maximum adsorption capacity obtained was 34 mg/g. Further, Yakkala et al. [91] derived biochar from buffalo weed by the pyrolysis method. The adsorption data were expressed via the “pseudo-second-order kinetic model.” The maximum adsorption capacity was 11.63 mg/g for Cd(ii). The principle mechanism behind the destruction of cadmium was “ion exchange and complexation.”

Tan et al. [92] examined the adsorption of Cd(ii) on the kiwi biochar functionalised with Fe–Mg-layered double hydroxide. They studied the impact of coexisting ions, pH, adsorbate dose, and contact time in the adsorption batch experiment. It was demonstrated that the adsorption capacity of this biochar was excellent. The maximum cadmium elimination capacity reached 25.6 mg/g.

Saeed et al. [93] extracted biochar from “rice husk” and examined the adsorption behaviour for Cd(ii). It was demonstrated that yield, along with the adsorption potential of biochar for Cd(ii) removal, was influenced by the time and temperature of pyrolysis. The authors have revealed that the optimised pyrolysis temperature was 4,580°C, reaction time of 2 h, and NaOH impregnation ratio of 3, leading to biochar yields of about 34.5 and 72% for the removal of cadmium and cadmium adsorption capacity of 17.8 mg/g.

Hussain et al. [94] created magnetically modified biochar out of used coffee grounds. This adsorbent had a very high capacity for Cd(ii) adsorption (10.42 mg/g). The characterisation of the biochar and modified biochar was performed by various approaches like XRD, BET, FTIR spectroscopy, and SEM. The “pseudo-second-order kinetic model” adequately explained the adsorption experiment and demonstrated that Cd(ii) adsorption occurred via chemisorption. “Langmuir and the Freundlich model” were applied to study the adsorption isotherm, and the data were well expressed using the “Langmuir model.” Adsorption of Cd(ii) via magnetic biochar was demonstrated to occur in a monolayer over a uniform surface. The thermodynamics study also revealed that the adsorption occurred spontaneously because the negative values of Δ*G* ^ο^ and positive values of *H* revealed that the adsorption process is endothermic.

Luo et al. [95] synthesised modified biochar by using corncob as a biowaste and the modification of the biochar was conducted by treating the corncob biochar with acrylonitrile. The modified biochar was shown to have an adsorption capacity of >85.65 mg/g for cadmium.

The roughness of the surface increases with increasing pyrolysis temperatures (BC350, BC450, and BC550), as shown by the SEM image of the sample. The MCB350 had a larger pore size, surface area, and pore volume, which were responsible for the “grafting reaction” in the “cellulose” of biochar and provided more Cd(ii) adsorption sites. While the rough surface vanished in BC450 and BC550, the pore size increased after the modification. The functional groups lying over the biochar were determined by FTIR spectroscopy. MBC350 has shown a broad band at 3,430 cm^−1,^ indicating the presence of the O–H bond, a vibration band near about 1,614 and 1,583 cm^−1^ confirming the presence of C═C and C═O groups (aromatic nature), and a peak at 2,249 cm^−1,^ revealing the presence of the C≡N group. Further, the authors have revealed the impact of the pH on the adsorption capacity of the different biochars. The elimination efficiency of Cd(ii) on the different biochars quickly increased from 2 to 4 and became constant at pH 5–7. MBC350 demonstrated greater than 90% removal efficiency for Cd(ii) at pH 3 compared to the other biochars; this may be due to the presence of cyano groups in MBC350 and the alkalinity of MBC350 after modification. An isotherm study was performed to explore the mechanism, and the data well fitted the “Langmuir isotherm” model, revealing that Cd(ii) adsorption was primarily caused by the ion exchange mechanism. The experimental data are well explained by the “pseudo-second-order kinetic model.”

### 5.2 Modified biochar

Khan et al. [105] created a “magnetic biochar composite” to recover Cd(ii) from water. The magnetic adsorption capacity was 46.90 mg/g (pH 6), and equilibrium was reached within 360 min. The authors also used the sorption isotherm model to determine the mechanism behind the adsorption of cadmium on magnetic biochar. The experimental data are well suited to the Langmuir isotherm model, which is associated with the adsorption process as a monolayer. The thermodynamic investigation shows that the process is “endothermic,” as Δ*G*° for the adsorption process is positive. Because precipitation is the primary reaction, the adsorption capacity increased with an increase in pH and decreased by >6.

Burk et al. [106] examined the adsorption of “cadmium” on the chitosan-coated gasifier biochar (CGBC) obtained from pine wood. The CGBC was synthesised by activation with aqueous CH_3_COOH and then treated with NaOH. It showed an adsorption capacity of 85.5 mg/g. The adsorption data fitted the Langmuir isotherm model and revealed that chitosan modification increased the adsorption capacity by producing amine coordination sites, which increased Cd(ii) adsorption. “Pseudo second-order” kinetic data well explained the adsorption data.

Jing et al. [107] created a clay–biochar composite from “Alternanthera philoxeroides (APBC)” and investigated its behaviour in the recovery of Cd(ii) from the aqueous phase. The maximum adsorption capacity was 44.3 mg/g (pH 6), and equilibrium was accomplished within 24 h. The isotherm study was conducted to explore the mechanism of Cd(ii) adsorption on APBC, and the data were well interpreted using the Freundlich isotherm model. The experiment was well expressed by the “pseudo-second-order kinetic model.” The thermodynamic analysis of the adsorption revealed that the process occurred spontaneously because of the negative value of the Gibbs free energy and occurred via physisorption.

Water supplies, human health, and the production of food are all at risk due to the frequent occurrence of Cd and Zn contamination in water. For the treatment of Cd^2+^ and Zn^2+^ in solutions, MnFeB, a new absorbent biochar modified with KMnO_4_ and haematite, was used. MnFeB has a higher adsorption capacity for Cd^2+^ and Zn^2+^ due to its rough surface structure, large specific surface area, larger total pore volume, huge functional groups, and abundant iron oxide. The maximal Cd^2+^ and Zn^2+^ adsorption capabilities of MnFeB in single metal systems were 1.88 and 1.79 times greater than those of unmodified biochar (CSB), respectively. In the binary metal system, the maximal Cd^2+^ and Zn^2+^ adsorption capabilities of MnFeB were 2.73 and 2.65 times greater than those of CSBs. The electrostatic contact, co-precipitation, π–π interaction, complexation, and ion exchange were important adsorption processes of Cd^2+^ and Zn^2+^ by MnFeB. Thus, water contaminated with Cd and Zn can be treated using MnFeB, a new absorbent [108].

In order to remove cadmium (Cd) from the contaminated water, this study used one-step and two-step methods to create rice straw biochar (BC), chitosan-modified rice straw biochar (CT-BC), and thiol-grafted chitosan-modified biochar (TH@CT-BC). Here, a two-step combination of chitosan and thiol was added to improve the biochar’s adsorption capacity. According to our findings, TH@CT-BC had the highest Cd adsorption at pH 5.5 (261.47 mg g^−1^), followed by CT-BC (103.14 mg/g) and BC (29.64 mg/g). In contrast to the Freundlich and Temkin models (0.949 and 0.925, respectively), the Langmuir and pseudo-second-order kinetic models (with *R* ^2^ values of 0.997 for TH@CT-BC) are best suited to the experimental data. In river water that had been tainted with 30 mg/L Cd, TH@CT-BC was still able to remove up to 89% Cd. Surface complexation and electrostatic interactions were shown to be the main underlying mechanisms for Cd elimination by TH@CT-BC in experimental studies and data computations. Furthermore, the vast volume of rice straw that was created must be used to create new materials that will aid in turning the trash into products that can be used to clean up the environment. As a result, our study shows that rice straw may be used to produce TH@CT-BC, an efficient Cd adsorbent from aqueous systems that may be investigated as a possible option for real-world wastewater treatment applications [109].

This study used a liquid-phase reduction approach to create a nano-zero-valent iron-loaded biochar–zeolite composite material (nZVI-BCZo) utilising biochar, zeolite, and FeSO₄·7 H₂O as precursors in order to remove Cd(ii) and As(iii) from water. The adsorption properties and performance of nZVI-BCZo for Cd(ii) and As(iii) were assessed by batch adsorption tests. Cd(ii) and As(iii) were found to have maximum adsorption capacities of 28.09 and 186.99 mg/g, respectively. The removal efficiency was more affected by pH than by temperature, and nZVI-BCZo had a greater affinity for As(iii) than for Cd(ii). Kinetic studies demonstrated that the adsorption process follows a monolayer adsorption mechanism and is mainly regulated by chemical adsorption. With adsorption efficiencies of 67.78% for Cd(ii) and 53.04% for As(iii), regeneration experiments showed that nZVI-BCZo maintained a good adsorption capacity after three cycles, suggesting its potential for recurrent use in water treatment applications. According to the economic analysis, the processing cost of nZVI-BCZo is more economical. Consequently, the study showed that nZVI-BCZo is a cost-effective, reusable, and effective adsorbent for treating water that contains heavy metals [110].

The effectiveness of pure biochar (BC) and silicate-modified biochar (SiBC) in Cd^2 +^ adsorption was examined in this work after they were made from sawdust by pyrolysis at 500°C. With a maximum Cd^2+^ adsorption capacity ranging from 87.19 to 179.85 mg/g, SiBC demonstrated competitive adsorption capability than BC. This is a significant increase over 4.13 mg/g recorded for BC. The batch experiment findings showed that a 1:1 ratio of Na₂SiO₃ to sawdust produced the best Cd^2+^ adsorption. The Freundlich isotherm model best explained the adsorption behaviour of Cd^2+^ on both BC and SiBC, whereas the pseudo-second-order kinetic model provided a good fit to the adsorption kinetics, indicating a combination of chemical and physical adsorption mechanisms.

Characterisation by XPS, FTIR spectroscopy, and XRD proved that complexation, ο-electron interactions, mineral precipitation, and cation exchange were involved in Cd^2+^ adsorption by SiBC. In addition to increasing the amount of silicate in biochar, silicate modification encouraged the development of alkaline groups (such as CO_3_^2−^ and ^−^OH). According to quantitative studies, the main adsorption mechanisms were precipitation and cation exchange, which accounted for 33.7–55.4% and 42.1–52.9% of the total Cd^2+^ adsorption for BC and SiBC, respectively. This study provides insights into sustainable environmental applications and highlights the possibility of modified sawdust-derived biochar for efficient heavy metal remediation [111]. Table 2 depicts the various biochar composites used for Cd(ii) recovery [112,113,114,115,116].

**Table 2:** Adsorption capacity of the biochar composite for Cd(ii)

Biochar as adsorbent	Pyrolysis temperature (K)	Initial concentration. (mg/L)	Adsorbent dose (g/L)	pH	Adsorption capacity (mg/g)	Isotherm model	Kinetic models	Contact time (min)	Mechanism	References
APB	573	30	0.5	6	44.3	Freundlich model	Pseudo-second	1,440	Physiosorption	[112]
MNPs/EDB/SS a	673	100	1	8	117.38	Freundlich model	Pseudo-second	120	Electrostatic binding	[113]
MBC	1,123			4–6		Langmuir	Pseudo-second			[114]
MgO–BCR	623	100	1		18.1	Langmuir	Pseudo-second		Surface complexation and electrostatic attraction	[115]
Mn/SA-BC@Nzvi	898	20			120	Langmuir	Pseudo-second	45	Surface complexation and precipitation	[116]

### 5.3 Mechanism for cadmium removal

The mechanisms controlling the “adsorption” of Cd(ii) via biochar are complexation, electrostatic interaction, and cation exchange. Harvey et al. [117] studied an adsorption mechanism responsible for “Cd(ii)” on a “biochar” extracted from loblolly pine, cordgrass, and honey mesquite. Biochar derived from these plants is categorised into two categories based on their capacity: higher and lower. The biochar derived from the plants is classified into two groups: one with a lower capacity and another with a higher capacity. The cation exchange mechanism was used to adsorb Cd(ii) by high-capacity group biochar. A flow calorimetric experiment was performed to investigate the behaviour of K and Cd(ii) to exchange Na-saturated biochar. According to flow calorimetry, the shape and duration of the cadmium heat signal for Na were the same as for K exchange Na; thus, “cation exchange” is the primary mechanism for Cd(ii) adsorption. Zhang et al. [118] demonstrated that the discharge of cations like K, Ca, Na, and Mg was similar to the degree of Cd(ii) adsorbed. The FTIR analysis of biochar after and before Cd(ii) adsorption revealed a negligible shift in carboxyl peaks, indicating that complexes with carboxyl groups were formed during Cd(ii) adsorption, which was an insignificant change.

## 6 Future scope and conclusion

Biochar is a sustainable adsorbent for the remediation of pollutants from wastewater, soil, and air. Another specific way to increase the use of biochar for eliminating specific contaminants is by activating it. Additional study is required to discover novel activation techniques as well as the adsorption and desorption mechanisms of different contaminants. A life-cycle analysis of biochar is required to assess its benefits to the environment and economy. New methodology breakthroughs have led to advancements in biochar characterisation methodologies. To achieve optimal efficiency, biochar characteristics and activation must be optimised. New technique adoption is influenced by accessibility and economic viability. Standard characterisation techniques must be used in light of the emergence of biochar as a substitute source in order to better understand its properties.

The level of heavy metal water contamination has risen to an alarming level. Cd(ii) is a highly toxic heavy metal that is harmful to plants and animals. As a result, recovering Cd(ii) from wastewater has become an urgent need for the entire universe. To recover Cd(ii) from water, various treatment methods like “floatation, filtration, ion exchange, and adsorption” have been used. Among these procedures, adsorption is very effective for remediating Cd(ii)-contaminated aqueous solutions. Further, multiple adsorbents were applied for the adsorption of Cd(ii), but there is a need for a sustainable adsorbent for the adsorption of Cd(ii),” and in this regard, biochar is the best adsorbent. This study has critically discussed the exploitation of biochar in the recovery of Cd(ii) from the aqueous phase.

Cadmium pollution is a major problem faced worldwide, and adsorption is one of the promising processes for its removal from wastewater. Activated carbon is used by many industries, but the cost is a major drawback of this adsorbent. Therefore, the increase in the use of bioadsorbent for the removal of heavy metals could be observed. According to the literature reviewed, bioadsorbents represent a promising green technology and can potentially be applied to full-scale wastewater treatment. The main objective of this review is to study toxicity due to cadmium exposure and to bring awareness to the public about the detrimental effects of cadmium on flora and fauna.

Future research would be directed towards the use of biochar in the treatment of real-world water. Moreover, research should be focused on the effect of other ions’ existence in the aqueous solution on the “capacity” of the biochar for the elimination of cadmium.
